# Role of presymptomatic transmission of COVID-19: evidence from Beijing, China

**DOI:** 10.1136/jech-2020-214635

**Published:** 2020-08-26

**Authors:** Yi Zhang, David Muscatello, Yi Tian, Yanwei Chen, Shuang Li, Wei Duan, Chunna Ma, Ying Sun, Shuangsheng Wu, Lin Ge, Peng Yang, Lei Jia, Quanyi Wang, Chandini Raina MacIntyre

**Affiliations:** 1 Beijing Center for Disease Prevention and Control & Beijing Research Center for Preventive Medicine, Beijing, China; 2 University of New South Wales, Sydney, Australia; 3 University of Nevada Las Vegas, Las Vegas, Nevada, USA; 4 Arizona State University, Tempe, Arizona, USA

**Keywords:** Communicable diseases, control of diseases, public health policy

## Abstract

**Background:**

The presymptomatic transmission of severe acute respiratory syndrome coronavirus 2 (SARS-CoV-2) has been documented in limited clusters, and it is predicted through modelling. However, there is a lack of evidence from observations with a large sample size.

**Methods:**

We used data from meticulous contact tracing of people exposed to cases of SARS-CoV-2 to estimate the proportion of cases that result from the presymptomatic transmission of the virus in Beijing during January 2020 and February 2020.

**Results:**

The results showed that presymptomatic transmission occurred in at least 15% of 100 secondary COVID-19 cases. The earliest presymptomatic contact event occurred 5 days prior to the index case’s onset of symptoms, and this occurred in two clusters.

**Conclusions:**

The finding suggested that the contact tracing period should be earlier and highlighted the importance of preventing transmission opportunities well before the onset of symptoms.

## BACKGROUND

The presymptomatic transmission of SARS-CoV-2 has been documented in single cases, family cases and other limited clusters.^1–5^ It is also predicted through modelling.^6^ However, there is a lack of evidence from observations with a large sample size. Using data collected through meticulous contact tracing from Beijing, China, we documented presymptomatic transmission in the contact tracing of 47 clusters.

## METHODS

We used data from the routine contact tracing of people exposed to cases of SARS-CoV-2 to estimate the proportion of cases that result from the presymptomatic transmission of the virus in Beijing during January 2020 and February 2020. Routine epidemiological response involved the careful contact tracing of all contacts of confirmed and suspected SARS-CoV-2 cases through face-to-face interviews that were conducted by the staff of the Center for Disease Prevention and Control (CDC) in the Beijing districts. The transmission exposure opportunity was identified during the investigation. Respiratory samples were collected and tested by RT-PCR. For the epidemiological investigation, data about the daily activities that were done, and the people who were in contact with each case, was aggregated from 14 days prior to the onset of symptoms to the day of isolation. Electronic payment history, receipts, travel tickets and so on were used to help the cases recall and verify their activities. In addition, contact tracing was augmented through the use of big data technology and mobile phone location data.^7^

According to the Chinese guidelines that govern contact tracing (versions 1–4), prior to February 21, only people who had contact with a case after showing symptoms or having a positive test result were considered as close contacts. Close contacts were requested to self-isolate at home or in designated quarantine facilities. The symptoms and body temperatures of close contacts were reviewed by healthcare staff by phone or in person at least twice a day. At the time of the investigation, all symptomatic contacts who were exposed during the index patient’s symptomatic period underwent SARS-CoV-2 testing. Close contacts were tested upon detection, regardless of whether they had symptoms or not. This was done so that potentially infected persons were detected early. However, testing of asymptomatic close contacts is not routine or standard procedure.

Clusters with a common index case were examined, and the epidemiological links of lab-confirmed cases in the clusters were determined by reviewing their contact history. Generally, cases with a travel history to Hubei or other provinces with recognised community transmission were considered as the index case of each cluster, while cases without such travel history or other risk factors were considered as secondary cases (local transmission). Community transmission in Beijing was contained well, and most reported cases in Beijing were linked to imported cases from Hubei or other provinces. Clusters that met the following criteria were included in this analysis:

1. The cases in the cluster were Chinese domestic cases.
2. There was at least one secondary case in the cluster.
3. All index and secondary cases that were included were PCR-confirmed.

Clusters were excluded if it was a nosocomial infection or a workplace cluster where the transmission chain could not be followed. Secondary cases were excluded if the time of the exposure could not be clearly defined. This investigation was undertaken in response to a public health emergency that was declared according to the laws of the People’s Republic of China on the prevention and treatment of infectious diseases. As part of the public health response, ethical approval was not required.

## RESULTS

**Figure 1:**
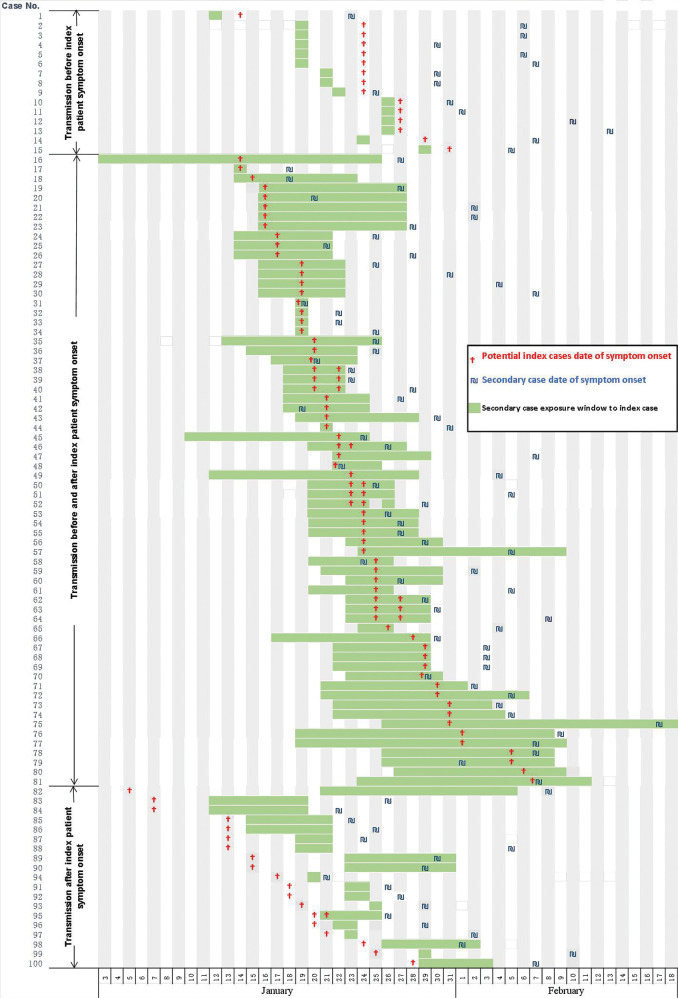
Exposure window for transmission to secondary case, and the onset date for the index cases and the secondary cases (If there are multiple potential index cases, the date of symptom onset of all potential index cases are marked).

From January 19, 2020 to February 29, 2020, a total of 91 clusters that involve 315 RT-PCR-confirmed domestic COVID-19 cases were identified in Beijing, excluding cases imported from other countries. Among them, 31 clusters involving 82 cases were excluded as there were no secondary cases in the cluster. Two clusters involving 49 cases were also excluded as the outbreaks happened in a hospital or company where the transmission chain cannot be identified. Eleven clusters involving 31 cases were excluded as the contacts are household members of the index cases whose starting date of exposure cannot be identified. In total, 47 clusters (53%) involving 100 secondary cases and 53 potential index cases were analysed in the study. Cluster size ranged between 2 and 8 cases, with a median of 3 cases. Among the 47 clusters, 23 (48.9%) had only one secondary case, and 24 (51.1%) had two or more secondary cases. The exposure window for the transmission to secondary cases and the onset date of the index and secondary cases are shown in figure 1. The onset date was defined as the first day in which fever or respiratory symptoms including sore throat, cough, diarrhoea or constitutional symptoms occurred.

Among the 100 secondary cases, 15 cases (15%) had contact with the index case before the index case showed symptoms (figure 1). All 15 of these secondary cases were exposed to the index case for only 1 day. For 12 cases, the contact event involved sharing a meal with the index case. For the remaining three cases, it involved having a conversation or a card game. The earliest presymptomatic contact event occurred 5 days prior to the index case’s onset of symptoms. This occurred in two clusters.

There were a total of 66 secondary patients who were exposed to the index patient before and after the index patient developed symptoms, with a median exposure interval of 8 days (IQR: 7–13). Among them, three secondary cases had an onset of symptoms before the index case did, and five secondary cases had an onset of symptoms on the same day as the index case. This suggests possible presymptomatic transmission.

There were 19 secondary patients who were exposed distinctly after the index case developed symptoms, with a median exposure interval of 8 days (IQR: 5–8).

## DISCUSSION

Our study demonstrated probable presymptomatic SARS-CoV-2 transmission in at least 15% of 100 secondary cases in 47 COVID-19 clusters. This is consistent with published cases and smaller cluster reports.^1–5^ A study based on the secondary analysis of 468 confirmed COVID-19 cases showed that at least 13% of the cases were exposed prior to the onset of symptoms.^8^ This is similar to our finding where 15% of secondary cases were exposed before the onset of symptoms in the index case. Moreover, considering that 66 secondary cases were exposed both before and after the index cases developed symptoms, our result indicates that the range of presymptomatic transmission may be from 15% to 81%. This is consistent with the range reported by the model from He *et al*, 2020, which estimates that 44% (95% CI 25% to 69%) of the cases are from presymptomatic transmission.^6^

The substantial proportion of presymptomatic transmission may in part be explained by the apparent silent transmission that has taken countries by surprise. This may shed some light on the degree by which presymptomatic transmission contributes to epidemic growth. This finding also supports the use of social distancing, face masks and proper hand hygiene to prevent the growth of the epidemic, even in the absence of symptoms.

In addition, among the 15 secondary cases who were exposed before the index case had an onset of symptoms, we found that 5 were detected through extended close contact tracing, while the other 10 were detected because the cases voluntarily sought medical attention. The result indicates that contact tracing needs to include a time window that lasts until the day before the onset of symptoms. In China, on February 21, 2010,^9^ the window for close contact tracing was adjusted to include 2 days before the onset of symptoms. However, the cases whose transmission occurred as early as 5 days ahead of the onset of symptoms in the index case suggest that the contact tracing period should be even earlier. At the very least, health instructions can be given to the contacts who were exposed to the index case even if they do not meet the criteria of close contacts. This would alert them with the risk and encourage them to self-quarantine and to actively report their health conditions.

This study has potential limitations. First, we were unable to determine the complete contact histories for all cases and clusters occurring in Beijing up to the end of February 2020. The epidemiological experience of index cases with a travel history and their contacts may not reflect the epidemiological experience of the general population during community transmission. Second, the lack of testing of all contacts during the index patient’s presymptomatic period, along with solely using the RT-PCR test, may result in an underestimation of the proportion of secondary cases as the reduction of viral shedding can be rapid. Third, although the study was conducted in Beijing early in the outbreak and widespread community transmission has not been detected there, it is possible that undetected community transmission may have explained the apparent presymptomatic transmission.

**What is already known on this subject**

- The presymptomatic transmission of SARS-CoV-2 has been documented in single cases, family cases and other limited clusters. It is also predicted through modelling. However, there is a lack of evidence from observations with a large sample size.

**What this study adds**

- Presymptomatic transmission occurred in at least 15% of 100 secondary COVID-19 cases and the earliest presymptomatic contact event occurred 5 days prior to the index case’s onset of symptoms. The finding suggested that the contact tracing period should be earlier and highlighted the importance of preventing transmission opportunities well before the onset of symptoms.
